# Targeting PI3K/mTOR Signaling in Cancer

**DOI:** 10.3389/fonc.2014.00084

**Published:** 2014-04-22

**Authors:** Alexandre Arcaro

**Affiliations:** ^1^Department of Clinical Research, Division of Pediatric Hematology/Oncology, University of Bern, Bern, Switzerland

**Keywords:** Akt, cancer, clinical trials, mTOR, phosphoinositide 3-kinase

The phosphoinositide 3-kinase (PI3K)/mammalian target of rapamycin (mTOR) pathway is very frequently activated in human cancer by a variety of genetic and epigenetic events. This pathway is thought to contribute to many of the hallmarks of cancer and a large array of agents targeting its key components are currently undergoing clinical testing in cancer patients. In addition to rapamycin analogs (“rapalogs”), which are approved for the treatment of multiple cancers, PI3K inhibitors are likely to be soon approved for B-cell malignancies (1, 2).

In this research topic, we have assembled a collection of articles describing recent key aspects of the role of the PI3K/mTOR pathway in cancer and the development of targeted therapies.

Martini et al. review the role of the different classes of PI3K isoforms as targets in oncology (3). Tzenaki and Papakonstanti focus on the role of the PI3K isoform p110δ in cancer (4). The role of the PI3K/mTOR pathway in cell cycle progression and metabolism is discussed by Vadlakonda and colleagues (5–7). Pardo and Seckl present an overview of S6K2, the p70 ribosomal S6 kinase homolog (8).

Porta and colleagues present an up to date overview of the development of selective inhibitors of Akt, mTOR, and PI3K with a focus on the latest clinical trials (9). Weigelt and Downward review the genetic determinants of response to these targeted agents (10). Fox et al. discuss the potential of co-targeting PI3K and the estrogen receptor (ER) in breast cancer (11).

## Conflict of Interest Statement

The author declares that the research was conducted in the absence of any commercial or financial relationships that could be construed as a potential conflict of interest.
